# In Situ Enhanced Raman and Photoluminescence of Bio-Hybrid Ag/Polymer Nanoparticles by Localized Surface Plasmon for Highly Sensitive DNA Sensors

**DOI:** 10.3390/polym12030631

**Published:** 2020-03-10

**Authors:** Seokho Kim, Bo-Hyun Kim, Young Ki Hong, Chunzhi Cui, Jinho Choi, Dong Hyuk Park, Sung Ho Song

**Affiliations:** 1 Department of Chemical Engineering, Inha University, Incheon 22212, Korea; seokho@inha.edu (S.K.); jinho@inha.edu (J.C.); 2 Division of Advanced Materials Engineering, Kongju National University, Chungnam 31080, Korea; bohkim@kongju.ac.kr; 3 Department of Physics, Gyeongsang National University, Jinju 52828, Korea; ykhong@gnu.ac.kr; 4 Department of Chemistry, College of Science, and Key Laboratory of Natural Resource of Changbai Mountains & Functional Molecules, Ministry of Education, Yanbian University, Yanji 133002, China; cuichunzhi@ybu.edu.cn

**Keywords:** surface plasmon, sensor, nanoparticle, photoluminescence, Ag, organic materials

## Abstract

We experimentally demonstrate the simultaneous enhancement of Raman and photoluminescence (PL) of core-shell hybrid nanoparticles consisting of Ag (core) and polydiacetylene (PDA, shell) through the assistance of localized surface plasmon (LSP) effect for the effective biosensor. Core-shell nanoparticles (NPs) are fabricated in deionized water through a sequential process of reprecipitation and self-assembly. The Raman signal of PDA on core-shell NPs is enhanced more than 100 times. Also, highly enhanced photoluminescence is observed on Ag/PDA hybrid NPs after coupling of the complementary *t*-DNA with *p*-DNA which are immobilized on PDA shell. This indicates that the core Ag affects the Raman and PL of PDA through the LSP resonance, which can be caused by the energy and/or charge transfer caused by the LSP coupling and the strong electromagnetic field near Ag NP surface. Only electrons present on the surface interact with the PDA shell, not involving the electrically neutral part of the electrons inside the Ag NP. Furthermore, this work shows that as prepared Ag/PDA NPs functionalized by probe DNA can sense the target DNA with an attomolar concentration (100 attomole).

## 1. Introduction

With the rapid development of optical spectroscopy instruments with high spatial resolution beyond the diffraction limit, the optical properties of individual nano-architectures have been successfully measured and analyzed [1,2,3]. As the size of materials goes down to or becomes less than the wavelength scale of visible light, the localized surface plasmon (LSP), which is a resonance oscillation of free or delocalized electrons at the nanomaterials that induces a strong electric field, becomes important for the analysis of non-linear optical properties. Many efforts for nanoscale optical characteristics, such as localized surface plasmonics (LSP), molecular nanophotonics, and opto-nano-bio-sensing, have been made by using a near-field scanning optical microscope (NSOM) and laser confocal microscope (LCM) in inorganic, organic, and bio-nanostructure [4,5,6,7,8,9,10,11,12]. The LSP observed from nanoparticles (NPs), nanotubes (NTs), nanowires (NWs), nanoshells (NSs), and nanocavities (NCs) made by metals, typically gold (Au) and silver (Ag), can significantly enhance the spectroscopic signals, such as absorption, fluorescence, and Raman effects [13,14,15]. Moreover, when the plasmon frequency of metal materials is resonated with the exciton of the organic materials in the hybrid nanostructure, the non-linearly enhanced photoluminescence or Raman signal can be observed even from single molecules [16,17,18,19,20,21,22]. This LSP resonant interaction with exciton occurring on the organic-metal hybrid system is believed to be due to the interaction of excited and delocalized electrons from organic materials, which are located between the interface of hybrid materials, with an enforced electromagnetic (EM) wave by the metal part [11]. Under the visible light irradiation, the free electrons in the surface of metal nanostructures are collectively oscillated by the EM field with a resonant frequency. As a result, an in-plane oscillation is trapped on the surface owing to the interaction with air or the free electrons of the dielectric materials. Therefore, the finely controlled hybrid nanomaterials, consisting of metal and/or organic luminescent molecules, can be a promising substance for the fundamental study of SP induced non-linear nano-optics [23,24,25]. So far, most of the research using LSP have utilized a single characteristic; for example, Raman effect or photoluminescence (PL), although some studies of simulations or bulky system experiments reported simultaneous enhancement of Raman and PL signals [26,27]. In situ observation of LSP enhanced Raman and PL signals from the hybrid nanomaterials still remains a challenge because of the overlapping signal range. Accordingly, the investigation of LSP enhanced Raman and PL from the hybrid NPs is an interesting subject not only for fundamental science such as nano-optics and nanotechnology, but also for advanced applications like displays and sensors [28,29].

Polydiacetylene (PDA) is one of the typical π-conjugated polymers that is mostly prepared using photopolymerization of self-assembled diacetylene (DA) monomers. Through 254 nm UV-light irradiation, the closely packed and properly ordered DA monomers undergo polymerization via 1, 4 addition reaction to form an alternating ene–yne polymer chain. As prepared PDA shows an interesting optical property of color change caused by the phase transformation from the initial blue to red upon environment stimuli such as temperature, pH, mechanical stress, and bio/chemical interaction. When a stimulus is applied to the PDA, the main chain is twisted by a functional group of PDA and a change in conjugation length occurs. Changes in the arrangement of electrons in the π-orbital result in different optical absorption and emission. Therefore, various sensors can be manufactured by adjusting the functional group at the ends of the DA monomer that receive stimuli [30]. Additionally, while the initial blue phase PDA exhibits negligible fluorescence originating from the relatively longer wavelength absorption, the red phase PDA absorbed in the shorter wavelength range shows the significantly higher intensity of red fluorescence [31,32,33,34,35]. It was also noted that both blue and red phase PDA is considered as Raman-active materials [36]. Therefore, when the PDA polymer hybridizes with metal NPs, an enhancement of the spectroscopic signals is expected due to the SP of metal. Recently, the surface plasmon resonance has been commonly utilized for the biosensors. Biosensors consist of a receptor that is specific to the analyte and a transducer that converts the interaction of recognizing moiety with analyte into the signal. Additionally, the biosensor can be divided into label type and label-free type according to the detection method, and the signal types are light emission or absorption, current, and color transformation. In particular, the importance of label-free type detection of very small amounts of DNA and biological tissue has grown significantly [37,38]. Emission biosensors require higher sensitivity because they should be recognized by optical signals [39,40,41]. Although there are several reports on biosensors using SP supporting biosensors, biosensors using PL and Raman as complementary sensing signals had not been reported until now.

Herein, we report on a biosensor using the enhanced Raman and PL spectra of the core-shell Ag/PDA hybrid NPs as complementary signals to detect DNA. We used the anthrax lethal factor DNA sequence as a *p*-DNA (NH2-5′-ATC CTT ATC AAT ATT TAA CAA TAA TCC-3′), with a concentration of 100 nM. For comparing the perfect match or not, we used two different *t*-DNA sequences. The complementary *t*-DNA and 1-mer mismatched *t*-DNA are listed as 3′-TAG GAA TAG TTA TAA ATT GTT ATT AGG-5′and 3′-TAG GAA TAG TTA TAA AAT GTT ATT AGG-5′, respectively. The biosensor system presented in this study can detect various substances depending on the *p*-DNA attached to the surface. Among them, anthrax, which is lethal to the human body, was chosen to verify the high selectivity of the sensor system. Toxins produced by the anthrax spores damage immune cells in the blood, causing shock and, in severe cases, acute death. The core-shell Ag/PDA hybrid NPs were fabricated by conventional reprecipitation and a hydrothermal process without additional chemical treatment on the metal surface. The measured Raman signals of the PDA NPs and the core-shell Ag/PDA hybrid NPs allows us to give reasonable estimates for the rates of the SP enhanced signal amplification. Additionally, the probe DNA was immobilized on the PDA layer of hybrid NPs. The structure of hybrid NPs was examined through high-resolution transmission electron microscope (HR-TEM) analysis. The simultaneous observations of the plasmonic effect on the Raman and PL, before and after injection of target DNA, were executed with high-resolution homemade LCM equipment. 

Furthermore, it may be explained that the Ag NPs coated with dual-phase PDA NS can act as an enhancement of the local electric field when excited by the appropriate incident wavelength light [42]. The energy matched core-shell hybrid nanostructures between Ag and PDA can contribute to a larger generation of electric field on the surface of the combined layers between the Ag NP and PDA NS, resulting in a significant enhancement of photo-physical signals such as Raman and PL. Due to the plasmon effect in the same single NP in the solid state, these signals could be obtained with a high resolution LCM system combined with commercial atomic force microscopy (AFM), which operates dynamically with small amplitude modulation [43]. 

## 2. Materials and Methods

### 2.1. Sample Preparation

The 10,12-pentacosadiynoic acid (PCDA) powder (purity 98%) was purchased from Sigma Aldrich Co. (Darmstadt, Germany) and used without further purification. The pristine PCDA NPs were prepared by a conventional reprecipitation method [39]. The PCDA powder was dissolved in tetrahydrofuran (THF) solvent with a concentration of 1 mg/mL, and then vigorously stirred for 10 min. The as-prepared PCDA solution was added into deionized (DI) water, forming spherical shaped NPs with a diameter of 170 (±45) nm. Before using for hybridization, this PCDA NPs solution was dissolved while stirring for 10 min in THF, and then ultrasonicated for 10 min. 

The spherical shaped Ag NPs were prepared by wet chemical reduction of silver nitrate (AgNO_3_, 25 mM, 2 mL, Sigma Aldrich Co., Darmstadt, Germany) with trisodium citrate (TSC, 2.2 mM, 500 μL, Sigma Aldrich Co., Darmstadt, Germany) based on DI water (18.2 MΩ, 16.5 mL). After finishing the reaction, Ag NPs were obtained by centrifuge. The as-prepared Ag NPs were dispersed in DI water again through the sonication process, in which Ag NPs were stably dispersed without additional chemical modifications. 

The core-shell Ag/PDA hybrid NPs were produced by utilizing hydrothermal action. Detailed experimental conditions and procedures were reported earlier [39].

### 2.2. DNA Hybridizations

The anthrax lethal factor DNA sequence was used as a *p*-DNA (NH_2_-5′-ATC CTT ATC AAT ATT TAA CAA TAA TCC-3′) with a concentration of 100 nM. To couple the *p*-DNA with the Ag/PDA core-shell NP and PDA nano-shell (NS), an amine group was attached at the 5′-end of the *p*-DNA sequence. All experiments were performed at room temperature. The *p*-DNA was diluted with deionized (DI) water (with the initial resistivity of 18 MΩ·cm Milli-Q water purifier). Then the immobilization of the *p*-DNA onto Ag/PDA core-shell NP and PDA NS was achieved through 10 min of stirring. The amine group (NH_2_) of DNA and the carboxyl group (COO–) of PCDA can lead to hydrogen bonding between the oxygen of the carboxyl group and the hydrogen of the amine group and hybridize the single-strand *p*-DNA with PCDA NS surface [44]. In order to remove the unreacted PDA, centrifuge within 1 min at 3000 rpm or less to obtain a residue.

The sequences for complementary *t*-DNA and 1-mer mismatched *t*-DNA are listed as 3′-TAG GAA TAG TTA TAA ATT GTT ATT AGG-5′and 3′-TAG GAA TAG TTA TAA AAT GTT ATT AGG-5′, respectively. Complementary *t*-DNA was used from 100 nM to 100 aM, and 1-mer mismatched *t*-DNA was used at 100 fM. The hybridization reaction of *t*-DNA was accomplished within 10 min in phosphate-buffered saline (PBS) stock buffer with pH = 7.4. The DI water was used to prepare buffer solutions from the PBS stock buffer. To measure LCM PL and Raman spectra under dry conditions, all samples were dried in a vacuum oven for 30 min.

### 2.3. Measurement

The growth confirmation of core-shell Ag/PDA NPs were investigated using a scanning electron microscope (SEM; JEOL JSM-5200, Tokyo, Japan) and a high-resolution transmission electron microscope (HR-TEM; JEOL, JEM-3010, Tokyo, Japan). The diameter of core-shell Ag/PDA hybrid NPs were calculated with the JEOL JSM-5200 measurement software. The simultaneous observation of the plasmonic effect on the Raman and PL, through recognition of target DNA for effective biosensors, were executed with high-resolution homemade LCM equipment. The nanoscale Raman and PL spectra for the Ag/PDA hybrid NPs were measured using a homemade LCM built around an inverted optical microscope (Axiovert 200, Zeiss GmbH, Oberkochen, Germany). The excited laser was controlled carefully during sample loading to avoid unnecessary irradiation and color transition. The 633 nm line of a He–Ne gas laser was used for Raman excitation. The 488 nm line of an unpolarized Ar ion laser was used for PL excitation. The spot size of the focused laser beam on the sample in the LCM experiment was estimated to be approximately 200 nm. The PL signal was focused on a multimode fiber that acted as a pinhole for confocal detection. The other end of the multimode fiber was connected to a photomultiplier tube for PL imaging for PL spectral measurements. The laser power incident was fixed on the sample, and the acquisition time was fixed for each PL spectrum in the LCM PL measurements at 10 μW and 1 s, respectively. Detailed experimental conditions were reported previously [39].

## 3. Results and Discussion

### 3.1. Ag/PDA Hybrid NPs and Probe DNA

**Figure 1:**
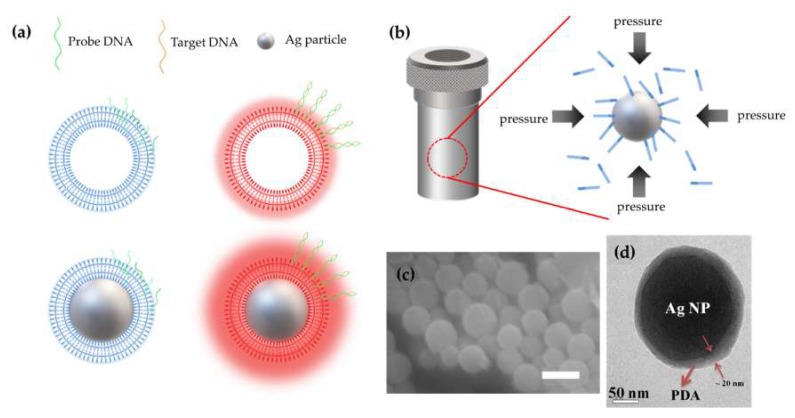
(**a**) Schematic illustration of the Ag (core)/PDA (polydiacetylene; shell) nanoparticles (NPs) with single-strand *p*-DNA which are immobilized onto the PDA shell (left) and after treatment of double-strand *p*-DNA/*t*-DNA (right). (**b**) Hybridization of PDA and Ag NPs via hydrothermal process. They were combined physically, attached by high pressure. (**c**) SEM image of core-shell Ag/PDA hybrid NPs (scale bar 200 nm). (**d**) Magnification of high-resolution transmission electron microscope (HR-TEM) image of core-shell Ag/PDA single NP.

During the hydrothermal process, high pressure (approximately 10 bar) was applied to PCDA and Ag NPs, and the as-prepared Ag NPs were coated by DA nano-shell (NS) as the carboxyl acid end-groups favor anchoring onto the surface of Ag NP. The alkyl of PCDA bonded with Ag NP and alkyl of other PCDA in the solution interact with each other to form a double layer. Finally, the outside of Ag/PCDA core-shell particles are surrounded by a carboxyl group (COO–). Moreover, the long alkyl chains stabilize the NS structure [40]. Figure 1a shows a schematic illustration of the DNA hybridization process using a single strand of Ag/PDA core-shell NP and PDA NS. The hydrogen from the amine group (NH_2_) at the end of DNA and the oxygen of the carboxyl group (COO–) at the end of PCDA form a hydrogen bond and combine single-strand *p*-DNA to the PCDA NS surface [44]. Complementary binding of *t*-DNA with *p*-DNA, which are bonded to the Ag/PCDA surface, twists the PDA backbone and alters the arrangement of π-electrons. This is the reason why they have different optical properties, such as absorption and color. The formation of the core-shell Ag/PDA hybrid NPs was visualized through a scanning electron microscope (SEM) and HR-TEM images, as shown in Figure 1b,c, respectively. The mean diameter of spherical shaped core-shell Ag/PDA hybrid NPs was estimated to be 150 (±30) nm, as shown in Figure 1b. From the magnified HR-TEM image of the single NP, the core-shell structure was clearly demonstrated, as shown in Figure 1c. The average thickness of the shell and mean diameter of the core in the NPs was estimated to be about 20 (±10) nm and 170 (±20) nm, respectively. 

### 3.2. Polymerization of PCDA into PDA

**Figure 2:**
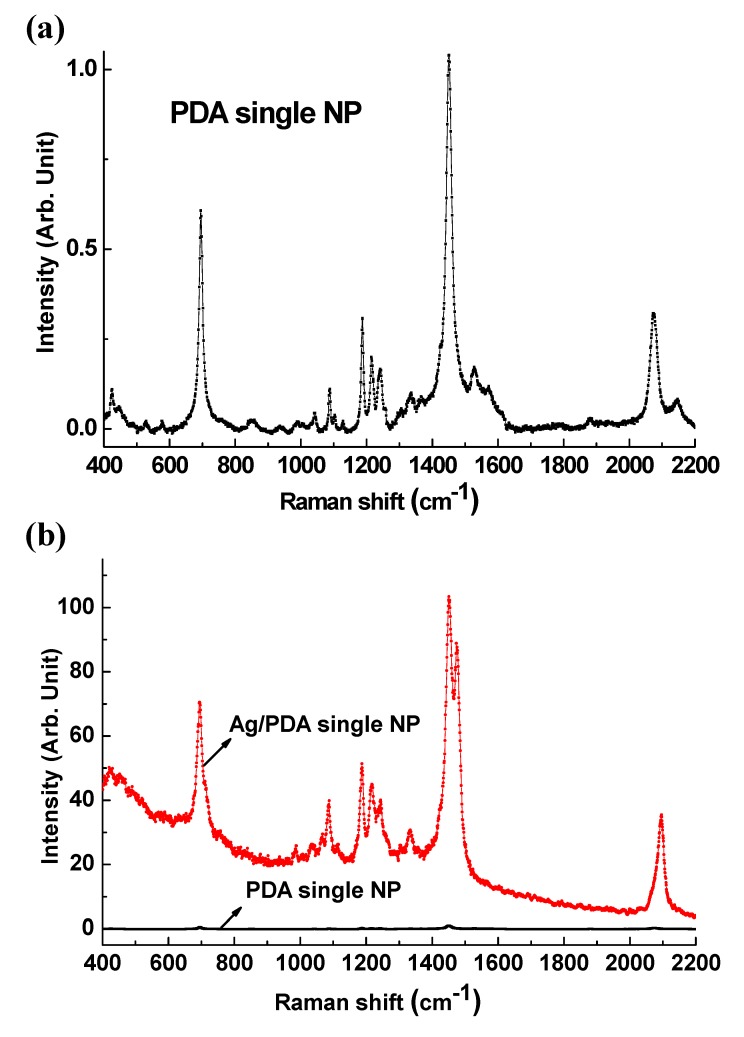
(**a**) LCM Raman spectra of the PDA single NP. (**b**) Comparison of the LCM Raman spectra of the PDA single NP and core-shell Ag/PDA hybrid single NP, respectively.

We measured the LCM Raman spectra for the PDA and core-shell Ag/PDA single NPs with the range of 400–2200 cm^−1^, as shown in Figure 2. Due to the experimental limit by grating used in the 633 nm excitation, we divided into two wavelength ranges and carefully measured the Raman signal. This allowed the bands of the C=C and C≡C stretching modes to be clearly discriminated. Figure 2a shows the Raman measured on a single PDA NP without core Ag, showing the characteristic vibration peaks for PDA structures. The signal intensity is normalized to the maximum peak at 1451 cm^−1^. The most prominent Raman characteristics peaks of the PDA were observed at 695, 1451, and 2072 cm^−1^, respectively, corresponding to the C–C, C=C, and C≡C stretching modes of the blue phase PDA molecules [35]. These characteristic peaks were observed at the exact same wavenumbers in the spectrum measured from the core-shell Ag/PDA single NP, as shown in Figure 2b. On the whole, the Raman scattering of the core-shell Ag/PDA single NP significantly increased compared to that of PDA NP. The signal intensity of Ag/PDA is normalized to the signal intensity of the peak at 1451 cm^−1^ in PDA NP. From the comparison, the intensities of C–C, C=C, and C≡C stretching modes in the Ag/PDA NP were enhanced about 83, 92, and 78 times, respectively, and the maximum enhancement ratio of signal intensity at 1451 cm^−1^ is over than 100. In addition, a shoulder peak with a wavenumber of about 1477 cm^−1^ was observed that was not observed in the spectrum of PDA NP. This can be attributed to the plasmon-induced Raman signal enhancement. The Ag NPs coated with dual-phase PDA NS are the reason for the enhancement of the local electric field when excited by the appropriate incident wavelength light [40]. The energy matched core-shell hybrid nanostructures between Ag and PDA can contribute to the generation of delocalized charges that induce the strong electric field on the surface of the Ag NP. This surface plasmon results in the significant enhancement of photo-physical signals such as Raman and PL. Using a high-resolution LCM system combined with a commercial AFM enables the measurement of weak signals from single core-shell Ag/PDA NP, even with small amplitude modulation [39]. The shoulder on the peak shown at 1400–1500 cm^−1^ is known to be observed at the intermediate purple state of PDA, indicating that the polymer backbone in the PDA shell layer has different microstructures. For example, blue and red phases, after grafting the *p*-DNA, although the blue phase is still dominant. However, it is also noteworthy that the probe DNA does not affect the backbone stretching vibration of PDA. From analysis based on previous reports, this contributed to the synthetic process in which the PCDA monomers were absorbed and fixed on the curved surface of Ag core NPs due to the anchoring effect, and they were photo-polymerized in the form of PDA NS onto the Ag NP. The PDA right above the Ag interface would experience difficulty in releasing lattice strain during the photo-polymerization. As a result, the backbone of the interfacial or inner PDA layer would be disordered and perturbed, leading to the minor red phase PDA. The variations of the Raman peaks position and intensity are listed in Table 1. 

### 3.3. LSP Induced PL Enhancement

**Figure 3:**
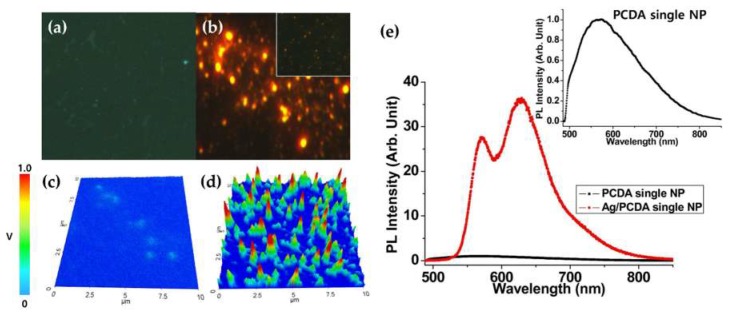
Color CCD images of (**a**) Ag/PDA/*p*-DNA NPs, (**b**) Ag/PDA/*p*-DNA+*t*-DNA NPs (The inset image is only *p*-DNA and *t*-DNA without Ag NPs), and (**c**,**d**) corresponding 3D PL images of Ag/PDA/*p*-DNA NPs and Ag/PDA/*p*-DNA + *t*-DNA NPs, respectively. The color scale bar on the left implies the LCM PL intensity of voltage. (**e**) LCM PL spectra of the PDA only single NP and core-shell Ag/PDA hybrid single NP. Inset: PL spectrum of the PDA only single NP for reference.

Figure 3 shows the luminescence color CCD images and the spectra of Ag/PDA, without and with coupling *p*-DNA to *t*-DNA. When immobilizing only *p*-DNA (Figure 3a), the luminescence color of the Ag/PDA core-shell hybrid NP was relatively weak green luminescence. However, when coupling *p*-DNA with *t*-DNA (Figure 3b), the emission color of the Ag/PDA NPs drastically varied from green to red, and the brightness was dramatically increased. It is well known that the red-shifted PL of PDA is due to the conformational change of the polymer main chain. Accordingly, it can be conjectured that the coupling of *t*-DNA to *p*-DNA induces the reduction of electrostatic force that causes the red-shifted PL of Ag/PDA NPs. It is known that both single-stranded and double-stranded DNA is negatively charged due to the phosphate group, however, the single-stranded DNA has more flexibility than the double-stranded DNA. Accordingly, the *p*-DNA, before coupling to *t*-DNA, can interact with the terminal amine group of the PDA through electrostatic force. The 3D LCM PL images of Ag/PDA/*p*-DNA NP (*p*-DNA-NP) and Ag/PDA/*p*-DNA + *t*-DNA NP (*p*-DNA-NP/*t*-DNA) were measured under identical conditions (Figure 3c,d). The mean voltages of the PL intensities measured from a single particle of *p*-DNA-NP and *p*-DNA-NP/*t*-DNA, were 23 ± 2 mV and 0.85 ± 0.12 V (error is standard error; SE and replicate number is 20), respectively. The 3D LCM PL images intensity of the functionalized *p*-DNA-NP/*t*-DNA was about 40 times brighter than that of the *p*-DNA-NP. The PL enhancement of *p*-DNA-NP/*t*-DNA is well matched to the result of CCD images shown in Figure 3a,b. Figure 3e shows the comparisons of the PL spectra of the *p*-DNA-NP to *p*-DNA-NP/*t*-DNA. For the quantitative analysis of the PL spectra, the maximum intensity of the LCM PL spectrum of the *p*-DNA-NP was chosen as the unit value for reference. The main PL peak of the *p*-DNA-NP was detected at about 570 nm. When coupling with *t*-DNA, we observed that the main PL peak of the *p*-DNA-NP/*t*-DNA was red-shifted to about 640 nm. The significant improvement in the PL efficiency of the bio-hybrid *p*-DNA-NP/*t*-DNA can originate from the plasmon assisted energy and/or charge transfer and strong local field enhancement effect between the Ag NP and PDA. This result suggests that the as-prepared *p*-DNA-NPs can be used as visible DNA detectors, through the strong enhancement and variation of luminescence color caused by the LSP of Ag NPs.

**Figure 4:**
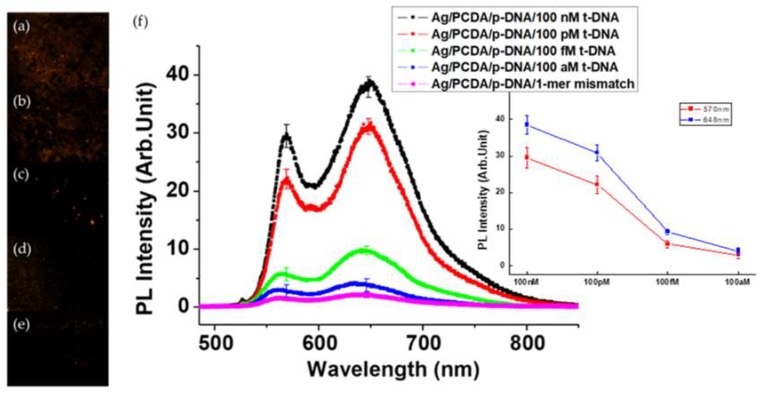
(**a**)–(**d**) Color CCD images of *p*-DNA-NP/*t*-DNA NPs depending on the concentrations of DNA from 100 nM to 100 aM and (**e**) 1-mer mismatched case. Scale bar: 10 μm. (**f**) Comparison of the PL spectra of *p*-DNA-NP/*t*-DNA depending on the concentrations of *t*-DNA (from 100 nM to 100 aM) and 1-mer mismatch case one (inset: PL intensity trend curve).

To see the sensitivity and selectivity of the *p*-DNA combined NP, we measured the PL intensity depending on the concentration of *t*-DNA and DNA with a mismatching sequence. Figure 4a–e show the interesting PL variation of *p*-DNA-NP/*t*-DNA as a function of the *t*-DNA concentration, varying from 100 attomole (aM) to 100 nanomole (nM), in which Figure 4e is the CCD image when the mismatching DNA was injected for comparison. With the increase in the concentration of *t*-DNA from 100 attomole (Figure 4d) to 100 nanomole (Figure 4a), the brightness of image increases and reaches a maximum at 100 nM. The two main LCM PL peaks of the *p*-DNA-NP/*t*-DNA are observed at ~570 nm and ~640 nm, respectively. To quantitatively compare PL intensity according to concentration, all of the data in this research has already removed the background data. The PL efficiency estimated by the integrated area under the spectrum line of *p*-DNA-NP/*t*-DNA with 100 nM of *t*-DNA is enhanced about forty-fold compared to that of the PDA only and *p*-DNA-NP. Table 2 lists the PL intensity at each peak corresponding to the concentration of *t*-DNA. Furthermore, the PL efficiency at 100 aM increases up to 4-fold, compared to that of *p*-DNA-NP without *t*-DNA and with 1-mer mismatch *t*-DNA. This demonstrates that our system has a potential to detect DNA with a single molecular level sensitivity and selectivity. This also supports the hypothesis we suggested in the above, that the single stranded DNA interrupts the energy and/or charge transfer from Ag NPs to the PDA main chain through electrostatic force. Although, it helps the conformational change of PDA from red phase to blue phase, whereas the coupling of *t*-DNA induces the decrease of flexibility of DNA that interacts less with the main chain of PDA. Thus, it is possible to maximize the efficiency of the plasmon effect of Ag NPs on the PL and Raman of PDA.

## 4. Conclusions

In summary, optically-direct DNA sensing was studied using Ag/PDA core-shell hybrid NPs with the help of the plasmon enhancement effect. For *t*-DNA recognized Ag/PDA core-shell hybrid NPs, we observed that the luminescence color of the PDA NPs varied from green to red, and a significant increase in PL efficiency in the dry conditions without using extra dyes, because of the plasmon enhancement effect. This PL enhancement was detectable at *t*-DNA concentrations ranging from 100 aM to 100 nM. This bio-hybrid nanosystem is also capable of discriminating a single-base mismatch against the perfect match of *t*-DNA. These results can open up a new area of investigation for the development of nanoscale label-free DNA sensors. 

## Figures and Tables

**Table 1:** Assignment and comparison of the Raman shift for the PDA only and Ag/PDA hybrid NPs.

	C–C Stretching Mode	C=C Stretching Mode	C≡C Stretching Mode
PDA NP (blue phase) (a)	695 cm^−1^	1451 cm^−1^	2072 cm^−1^
Core-shell Ag/PDA NP (b)	695 cm^−1^	1451 cm^−1^ (blue phase), 1477 cm^−1^ (red phase)	2076 cm^−1^
Enhancement Ratio of Intensities (case b/case a)	83	92	78
Normalized LCM Raman Intensity wrt C=C	0.51	1.0	0.35

**Table 2:** LCM PL intensities of Ag/PDA/*p*-DNA + *t*-DNA NPs with various concentrations of *t*-DNA.

	100 nM	100 pM	100 fM	100 aM
Intensity at 570 nm (a. u.)	29.5	22.1	5.9	2.9
Intensity at 648 nm (a. u.)	38.5	30.8	9.3	4.0
